# Effect of Covalent Functionalization on Thermal Transport across Paraffin/Graphene Nanocomposite Interfaces

**DOI:** 10.1021/acsomega.4c11293

**Published:** 2025-04-15

**Authors:** Xuehong Wu, Xufeng Lang, Zhijuan Chang, Mengyao Liu, Wenfeng Hu, Yong Liu, Cai Lv

**Affiliations:** † School of Energy and Power Engineering, Zhengzhou University of Light Industry, Zhengzhou, Henan 450002, China; ‡ Henan International Joint Laboratory of Energy Efficient Conversion and Utilization, Zhengzhou, Henan 450002, China; § Key Laboratory of Cold Chain Food Processing and Safety Control (Zhengzhou University of Light Industry), Ministry of Education, Zhengzhou, Henan 450002, China

## Abstract

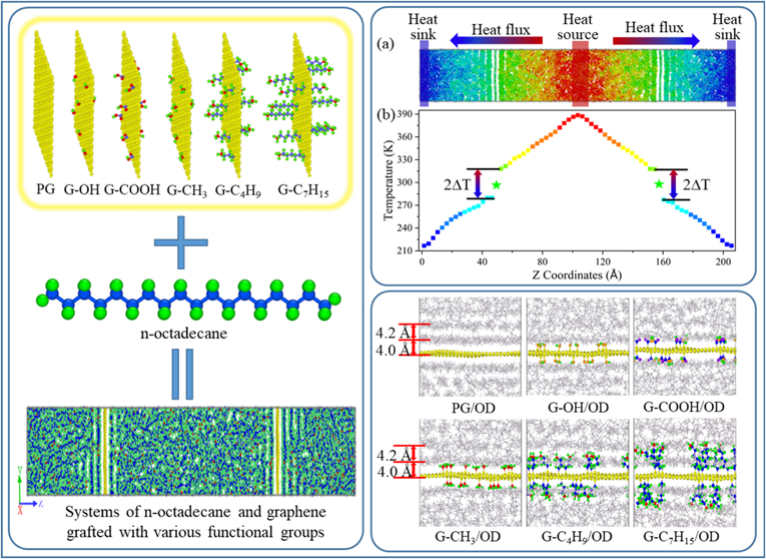

The low interfacial heat transfer efficiency limits the thermal conductivity (TC) of the paraffin/graphene composite phase change material. In this study, *n*-octadecane is used to represent paraffin. The interfacial thermal conductance (ITC) and overall thermal conductance (OTC) of pristine graphene/*n*-octadecane (PG/OD) and graphene/*n*-octadecane (G/OD) grafted with hydroxy (−OH), carboxyl (−COOH), methyl (−CH_3_), butyl (−C_4_H_9_), and heptyl (−C_7_H_15_) are explored using nonequilibrium molecular dynamic simulation methods. The results show that alkyl functional groups can significantly improve ITC and OTC, and the improvement effect increases with the increase of chain length. However, −OH and −COOH only slightly increase ITC, and −OH actually decreases OTC. At the grafting density of 0.01497 Å^–2^, –C_7_H_15_ increases the ITC and OTC by 128 and 34%, respectively. The phonon density of states analysis explains that –C_7_H_15_ demonstrates the greatest effect in enhancing interfacial thermal coupling, followed by –C_4_H_9_, −CH_3_, −COOH, and −OH. Moreover, the long-chain alkane functional groups may improve the interlayer thermal conductance of the near-wall layer, and thus the OTC. Finally, according to the calculated ITC and effective medium theory, the TC of G/OD composites is predicted. This work provides valuable guidance for exploiting the potential of the TC of paraffin/graphene composites.

## 1 Introduction

Solid–liquid phase change energy storage technology enables the storage and release of heat, making it widely applicable in areas such as industrial waste heat recovery, solar thermal energy storage, and electronic device thermal management.^1^ The key to this technology lies in the performance of phase change materials (PCM). Organic PCM represented by paraffin are commonly used as matrix of PCM due to their nontoxicity, noncorrosiveness, and chemical stability.^2,3^ However, the low thermal conductivity (TC) of paraffin limits its energy storage efficiency. In practical applications, high TC fillers are often introduced to improve the TC.^4,5^

Paraffin, as a promising polymer-based organic PCM and thermal interface material, shows inherently low thermal conductivity typically ranging between 0.1 and 0.4 W/(m·K).^6^ To improve this, graphene, known for its high specific surface area and TC, is often introduced into the paraffin matrix.^7,8^ Goli et al.^9^ showed that thermal management and the reliability of Li-ion batteries can be drastically improved using hybrid phase change material with graphene fillers. Mehrali et al.^10^ synthesized a form-stable composite PCM prepared by paraffin and graphene oxide (GO) sheets. The TC of paraffin in the molten state and solid state is only 0.305 and 0.287W/(m·K), respectively. The TC of paraffin/GO composites can increase to 0.985 W/(m·K) and 0.932 W/(m·K) in the corresponding state, respectively. A recent study introduced the graphene film into graphene foam using the ice-template method, resulting in a composite with paraffin wax that achieved a TC of 11.594 W/(m·K), which is 44 times higher than that of bulk paraffin, with a mass fraction of only 1.14 wt %.^11^ Despite these advancements, the TC of paraffin/graphene composites still has great potential for improvement due to the excellent thermal conductivity of graphene.^12−14^ The worse-than-expected TC of composites is attributed to the high interfacial thermal resistance (ITR, or Kapitza resistance) between paraffin matrix and graphene fillers.^15^ Therefore, it is necessary to study the internal mechanism of reducing the ITR of paraffin/graphene composites, so as to provide theoretical guidance for experimental research on exploiting the potential of TC of paraffin/graphene.

The methods for studying interfacial heat transfer generally include experimental method and theoretical computational method. Experimental methods include the time-domain thermoreflectance, the thermal bridge method, the 3ω method, and the electron-beam self-heating method. The most popular computational methods include lattice dynamics, molecular dynamics (MD), the Green’s function method, and the Boltzmann transport equation method.^16^ Experimental methods often necessitate specific requirements regarding the material’s shape, size, structure, etc., as well as testing conditions, which demand expensive experimental equipment, leading to higher costs. When it comes to nanoscale studies, experiments are often unable to capture the interatomic interactions.^17^ Computational methods can simulate complex physical processes with fewer requirements on experimental conditions and material properties, and are more cost-effective.^18^ Additionally, computational methods can investigate the intrinsic mechanisms of interfacial heat transfer theoretically.^19,20^ Among these, MD simulations can be categorized into nonequilibrium molecular dynamics (NEMD) and equilibrium molecular dynamics (EMD) simulations. NEMD simulation method can directly probe the interfacial temperature jump, and therefore, this method has been widely applied in the study of interfacial heat transfer in composites.^21−23^

Reducing the ITR can be achieved through the control of both external and internal conditions. For the former, it primarily involves applying pressure, controlling temperature, and applying physical fields. For the latter, it primarily encompasses adjusting the roughness of the interface, performing covalent or noncovalent functionalization at the interface, and introducing defects. These methods all intend to achieve tight bonding at the interface or improve the phonon vibration matching of interfacial materials, thereby reducing the ITR.^24^ Among these methods, both the noncovalent functionalization and covalent functionalization are effective in reducing the ITR of graphene/polymer composites. In the noncovalent functionalization method, there is no chemical bond between the functional molecules and graphene; the interaction is purely van der Waals. Lin and Buehler^25^ introduced the noncovalent molecule alkyl-pyrene on graphene surface to study the effect of noncovalent functionalization on the interfacial thermal conductance (ITC) of graphene-octane composite using MD simulation. They found that the best linker candidate enhances the ITC of graphene/organic composites by about 22%, which is attributed to its capability to compensate for the low-frequency phonon mode of graphene. Wang et al.^26^ systematically investigated the effect of noncovalent functional molecules such as 1-pyrenebutyric acid, 1-pyrenebutyl, and 1-pyrenebutylamine on the ITR of paraffin/graphene using MD simulation. The results indicated that the ITR of paraffin/graphene composites is reduced by 16–17% when the functional molecular mass ratio is 26–28%. Due to the stronger interaction of covalent bonds, covalent functionalization is able to enhance the thermal transport across paraffin/graphene interfaces more effectively than noncovalent functionalization. Cui et al.^27^ investigated the thermal transport between single-layer GO and water through transient MD simulations, which mimic the laser heating of graphene in water, as well as NEMD simulations. They found a nearly 1 order of magnitude increase in the ITC of graphene/water with the addition of functional groups (hydroxyl and epoxide) to the graphene surface. Yang and Cao^28^ carried out NEMD simulation to study the influence of epoxy and hydroxyl groups on ITC of graphene/calcium-silicate-hydrate (C–S–H). They found that the ITC values of GO-hydroxyl/C–S–H and GO-epoxy/C–S–H at 20% of the O concentration are 46.9 and 12.8 times higher, respectively. Wang et al.^29^ found that introducing covalent functional groups can significantly reduce the ITR of paraffin/graphene. With a 5.36% coverage of butyl, the ITR of paraffin/graphene is reduced by 56.5%. Liu and Zhang^30^ found that introducing carboxyl groups can significantly reduce the ITR of graphene/octadecanol nanocomposites and the generation of hydrogen bonds enhances the interface thermal coupling. Wang et al.^31^ investigated the effect of the introduction of amino groups on the ITR between epoxy and graphene edges using NEMD simulations. The results show that the active amino groups reduce the ITR to 42%. These studies investigated the enhancement effects of covalent functionalization and noncovalent functionalization on the ITC of paraffin/graphene and their internal mechanism at a theoretical level.

These studies provide insights into covalent functionalization strategies for improving the ITC of paraffin/graphene composites. However, the thermal transport process at the paraffin/graphene interface is complex. In the current literature, there has been insufficient exploration of the comprehensive effects of functional group types, alkyl functional group chain length, and grafting density on heat transfer at the paraffin/graphene interface. Further systematic and in-depth investigations are needed to find specific covalent functionalization strategies that can more clearly guide experimental design.

In this paper, NEMD simulations are performed to systematically study the enhancement effects of the covalent functional group type, chain length of alkyl functional group, and grafting density on thermal transport across graphene/paraffin nanocomposite interfaces. The ITC and overall thermal conductance (OTC) of the selected volumes are investigated. First, the ITC values of paraffin/graphene grafted with different covalent functional groups and varying graft densities are calculated. Meanwhile, the phonon density of states (PDOS) is calculated with the aim of further exploring the internal mechanism responsible for the effect of functionalization on the ITC. Second, the OTC values of graphene/paraffin are calculated, followed by an analysis of the effects of covalent functionalization and the molecular distribution of paraffin matrix near the interface on the OTC. Finally, based on effective medium theory (EMT) and the above calculation results, the TC values of graphene/paraffin composites composed of graphene grafted with different covalent functional groups and paraffin matrix are predicted.

## 2 Computational Methods

### 2.1 Initial Configuration of Graphene/Paraffin

Paraffin is a complex mixture composed of various *n*-alkanes. *N*-Octadecane, with its well-defined chemical composition and properties, physical characteristics similar to those of paraffin, and excellent qualities as a phase change material, is extensively utilized in experimental and simulation studies. Therefore, *n*-octadecane (OD) is chosen as a simplified model for paraffin due to its general thermodynamic properties.

**Figure 1:**
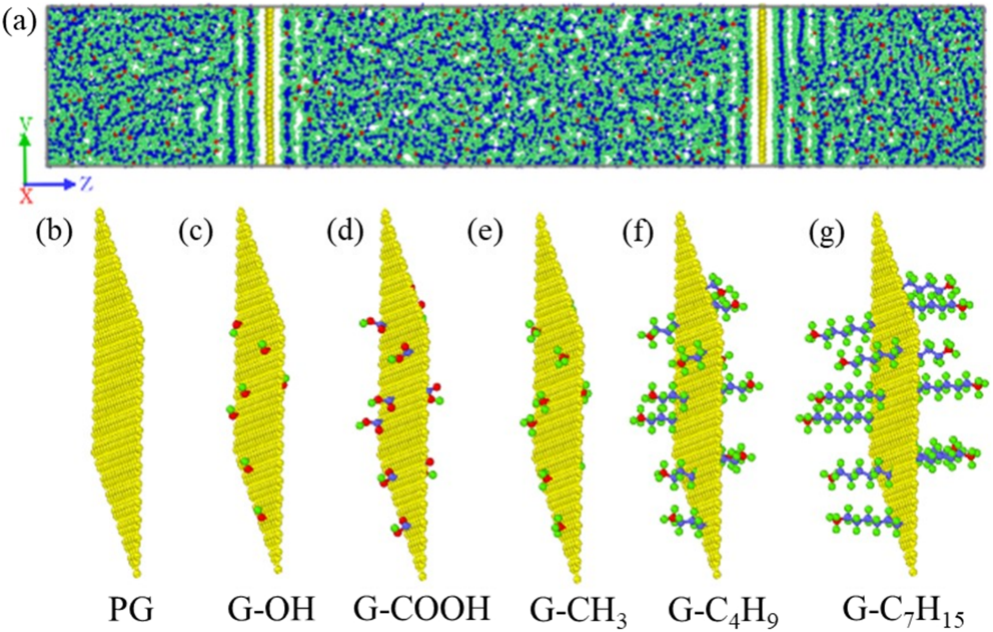
Structural models of (a) PG/OD, (b) PG, (c) G–OH, (d) G-COOH, (e) G-CH_3_, (f) G-C_4_H_9_, and (g) G-C_7_H_15_.

Figure 1 presents the structural models of the *n*-octadecane/graphene composite system and graphene grafted with various functional groups. As shown in Figure 1(a), the model of G/OD nanocomposites consists of 420 *n*-octadecane molecules and two graphene sheets. Periodic boundary conditions are applied in all three directions. After quenching and sufficient relaxation, the model attains dimensions of 34.9 Å × 34.5 Å × 205.7 Å at 300 K. Luo and Lloyd^32^ found that when the cross-sectional width exceeds 19.68 Å and the length of paraffin block is greater than 35 Å, the ITC exhibits insensitivity to model size. This study focuses on the effect of covalent functional groups on thermal transport across paraffin-graphene nanocomposite interfaces. Based on previous studies,^29,30,32,33^ the model size adopted in the present study is deemed reasonable. Figure 1(b–g) shows the structure of pristine graphene (PG), hydroxy-functionalized graphene (G–OH), carboxyl-functionalized graphene (G-COOH), methyl-functionalized graphene (G-CH_3_), butyl-functionalized graphene (G-C_4_H_9_), and heptyl-functionalized graphene (G-C_7_H_15_). These covalent functional groups are randomly grafted onto both sides of the graphene. The grafting density is defined as σ = *N*/*A*, where *N* is the number of covalent functional groups and *A* is the surface area of graphene.^34^ A grafting density that is too low would fail to significantly enhance interfacial heat transfer, whereas an excessively high grafting density would lead to a reduced mass fraction of graphene sheets themselves, potentially impeding the composite material from achieving the desired thermal conductivity. Therefore, the grafting densities adopted in the present study are 0.00499, 0.00998, and 0.014970 Å^–2^, which are commonly used grafting densities in experimental and simulation studies.^35^

### 2.2 Force Fields

The Tersoff potential^36^ is extensively used in simulating the thermal properties of graphene. The polymer consistent force field (PCFF)^37^ has been employed to investigate the thermal transport properties in polymeric materials, and the obtained results are in excellent agreement with experimental results. Based on previous research, the Tersoff potential for graphene and the PCFF force field for polymer have achieved remarkable results in calculating the ITC of graphene/polymer nanocomposite.^29,30,33,38^ Therefore, in the present study, the Tersoff potential with Lindsay and Broido modification^39^ is used to describe the interaction between the carbon atoms in graphene. The *n*-octadecane molecules and covalent functional groups are described by the PCFF force field, which obtained excellent results in previous studies. The interactions between covalent functional groups and their bonded carbon atoms in graphene are described by the PCFF force field. The long-range Coulombic interactions are calculated using Particle–Particle-Particle-Mesh (PPPM) solver with an accuracy of 10^–5^. The PPPM solver maps atom charge to a 3D mesh, uses 3D FFTs to solve Poisson’s equation on the mesh, and then interpolates electric fields on the mesh points back to the atoms. The van der Waals (vdW) interactions between *n*-octadecane and graphene, *n*-octadecane and covalent functional groups, as well as covalent functional groups and their nonbonded carbon atoms in graphene are described by the Lennard-Jones (LJ) potential with applying the Lorentz–Berthelot mixing rule. The cutoff radius is set to 14.0 Å. The LJ potential is expressed as
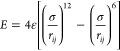
1where *r*_*ij*_ represents the distance of atoms; ε represents the energy constant; and σ represents the distance constant. The LJ parameters ε = 0.00239 eV and σ = 3.412 Å are used for the atoms in graphene.^40^ The detailed LJ potential parameters^29^ are given in Table 1.

**Table 1:** LJ Potential Parameters Used in the Simulations

atom types	energy constant ε (eV)	distance constant σ (Å)
carbon in graphene	0.002390	3.412
carbon in *n*-octadecane or alkyl	0.002342	4.010
carbon in carboxyl	0.005204	3.810
oxygen single bonded to carbon in carboxyl	0.010407	3.420
oxygen double bonded to carbon in carboxyl	0.011578	3.300
oxygen in hydroxyl	0.010407	3.535
hydrogen bonded to carbon	0.000867	2.995
hydrogen bonded to oxygen	0.000564	1.098

### 2.3 Simulation Details

**Figure 2:**
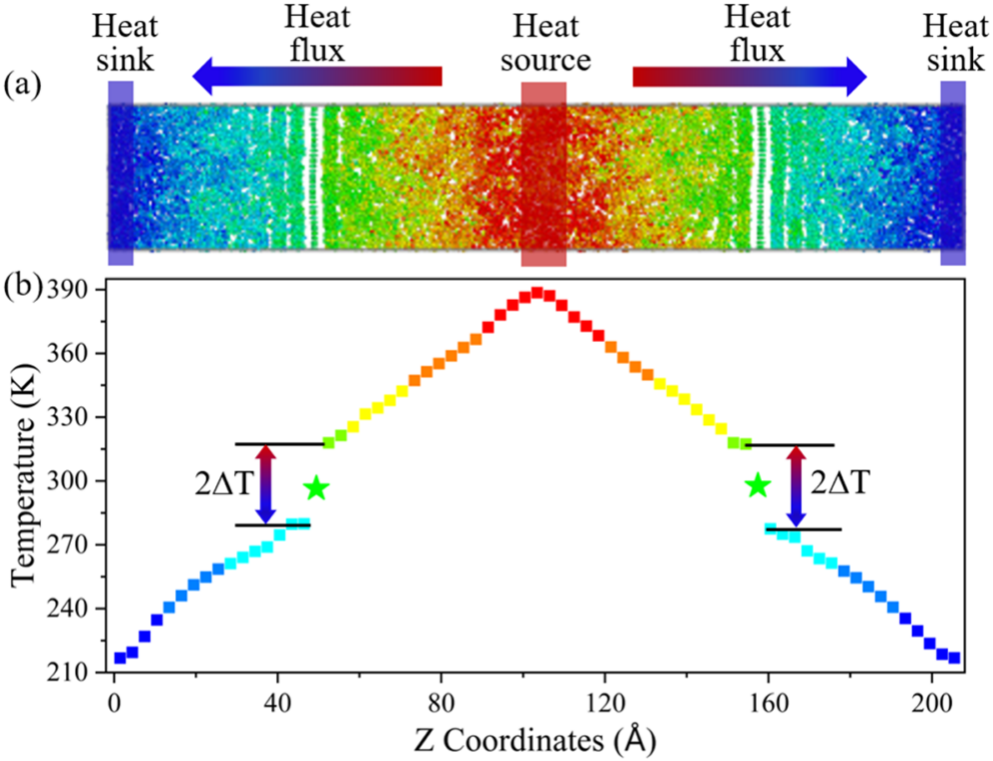
(a) Schematic diagram of heat transfer in the G/OD nanocomposite system. (b) Steady-state temperature gradient along the heat flux direction.

The Large-scale Atomic/Molecular Massively Parallel Simulator (LAMMPS)-22Oct2020 molecular dynamics package^41^ is used to perform all the MD simulations. The LAMMPS version used in this study is LAMMPS-29Oct2020, and packages such as CLASS2, KSPACE, and MANYBODY are utilized. The computer is equipped with two Intel Xeon Platinum 8375C processors, amounting to a total of 64 cores. The ITC of the G/OD is calculated using the NEMD method. The time step is 0.25 fs. At the beginning of the simulation process, the conjugate gradient algorithm is used to minimize the energy of the initial structure model. After that, the simulation process mainly consists of the following processes: First, the system is fully relaxed at 400 K and 1 atm for 2 ns in the NPT ensemble and then rapidly cooled to 300 K at a cooling rate of 0.4 K/ps. Thereafter, the system is fully equilibrated at 300 K and 1 atm for 0.5 ns in NPT ensemble and then switched to NVT ensemble at the same temperature for 0.5 ns to reach an equilibrium state. Finally, the system is switched to NVE ensemble to ensure energy conservation, and the ITC of G/OD is calculated using the NEMD method. In this method, a stable temperature gradient is established in the simulation box by defining regions of the heat source and heat sink. Figure 2(a) shows the schematic of the algorithm to evaluate the ITC of G/OD. The middle and two ends of the composite model are divided as the heat source and sink regions, respectively. A constant heat flux *q* for 4 ns is imposed between the heat source and sink regions under NVE ensemble to ensure the temperature gradient reaches a steady state. The heat imposed per unit time, denoted as *Q*, is 8.0 kcal/(mol·ps). In this paper, the cross-sectional area *S*_a_ of the model is about 34.9 Å × 34.5 Å. Therefore, the heat flow *q* can be obtained by *q* = *Q*/2*S*_a_.The steady-state temperature gradient along the heat flux direction is presented in Figure 2(b). It is evident that the temperature changes approximately linearly along the direction of the heat flux, while there is a temperature jump of Δ*T* near both sides of the graphene interface. Thereafter, the ITC(*G*_i_) can be evaluated by the following expression
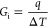
2where Δ*T* is obtained by averaging the data collected over a period of 2 ns in the steady state. The ITC of the G/OD is then obtained. The error bars are calculated using the block averaging approach.^42^

## 3 Results and Discussion

### 3.1 Validation of Models and Methods

To validate the models and methods employed in the present study, the TC of bulk OD with dimensions of approximately 32.5 Å × 32.5 Å × 99.8 Å is calculated using Fourier’s law, which is expressed as κ = *J*/(∂*T*/∂*z*), where *J* represents heat flux and ∂*T*/∂*z* represents the temperature gradient along the heat flux direction. The calculated TC of bulk OD is 0.169 ± 0.001 W/mK, which is in good agreement with the experimental^43,44^ and simulated^30,45^ results. As shown in Figure 2(b), the Δ*T* of PG/OD with the size of 34.9 Å × 34.5 Å × 205.7 Å calculated by NEMD simulations is finally determined to be 19.65 K. Therefore, the ITC of PG/OD is 117.72 MW/m^2^K, which is very close to reported ITC values for polymer/pristine graphene.^30,33,35,46,47^ These comparative results confirm the validity of the models and methods, which should be reasonable to study the thermal transport properties across paraffin/graphene nanocomposite interfaces.

### 3.2 Effect of Covalent Functionalization on the ITC of G/OD

**Figure 3:**
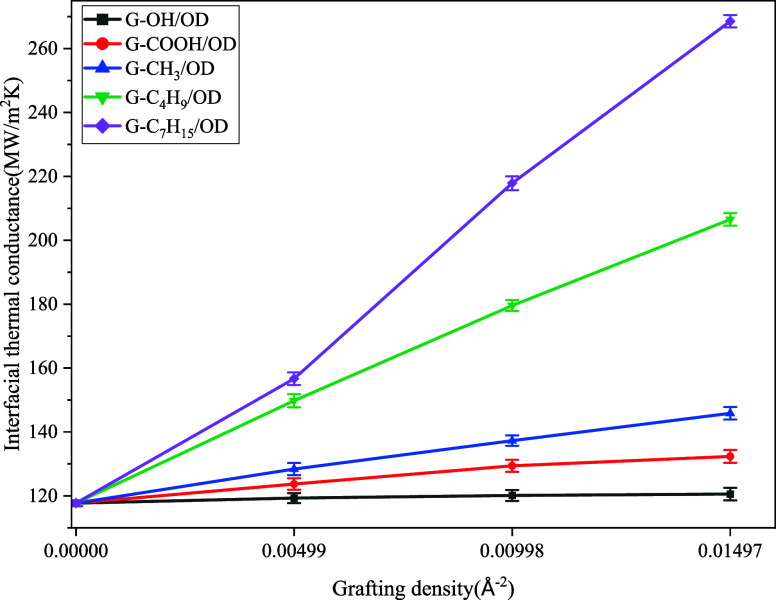
Variation of the ITC of G/OD grafted with various functional groups with respect to grafting density.

With the ITC of PG/OD as a reference, the variation of ITC of G/OD grafted with various functional groups with respect to graft density, as shown in Figure 3, provides a clear comprehension of the effect of functional group type, chain length of alkyl functional groups, and grafting density on ITC of G/OD. It is apparent that the ITC of G/OD increases with the increase in graft density. Interestingly, there are obvious differences in the enhancement effect of various functional groups on ITC. The ITC values of G/OD grafted with −OH and −COOH functional groups do not significantly improve. Even at a higher grafting density (0.01497 Å^–2^), the ITC of G–OH/OD and G-COOH/OD only increase to 120.57 and 132.35 MW/m^2^K, which are only 1.03 and 1.13 times that of PG/OD, respectively. In contrast, the alkyl functional groups significantly improve the ITC of G/OD, and this improvement increases with the chain length. At a lower grafting density (0.00499 Å^–2^), the ITC values of G-CH_3_/OD, G-C_4_H_9_/OD, and G-C_7_H_15_/OD are 128.39, 149.78, and 156.68 MW/m^2^K, respectively. While the ITC values of C_4_H_9_/OD and G-C_7_H_15_/OD are close at this grafting density, the enhancement effect of –C_7_H_15_ on the ITC of G/OD increases rapidly with a higher grafting density. When the graft density increases to 0.01497 Å^–2^, the ITC values of G-CH_3_/OD, G-C_4_H_9_/OD, and G-C_7_H_15_/OD increase to 145.85, 206.51, and 268.56 MW/m^2^K, respectively. This indicates that the interfacial thermal coupling between G-C_7_H_15_/OD is significantly enhanced as the grafting density increases. If –C_7_H_15_ is used to improve the TC of G/OD, a grafting density of 0.00998 Å^–2^ or higher is recommended.

As suggested by the theoretical models of interfacial heat transfer,^48^ the inherent limitation of ITC is dictated by the overlap of the PDOS between the two interfacing materials.^49^ The greater the overlap of PDOS, the higher the ITC. To elucidate the underlying mechanisms for the effect of various covalent functional groups on the ITC of G/OD, we calculated the PDOS in the frequency domain and a correlation factor *S* to reflect the phonon matching rate. The PDOS for pristine graphene, functionalized graphene, and *n*-octadecane are obtained by performing the Fourier transform of the normalized velocities autocorrelation function of atoms
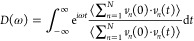
3where ω represents frequency, *N* represents the number of atoms, and *ν*_*n*_(0) and *ν*_*n*_(*t*) refer to velocities of atom *n* at initial time and at time *t*, respectively. In this paper, the different components of the system, i.e., *n*-octadecane, graphene, or functionalized graphene, are normalized, respectively.

The overlap between two PDOS spectra is quantitatively evaluated a correlation factor *S*,^50^ and *S* is defined as
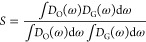
4where *D*_O_ represents the spectra of *n*-octadecane and *D*_G_ represents the spectra of PG or functionalized graphene.

**Figure 4:**
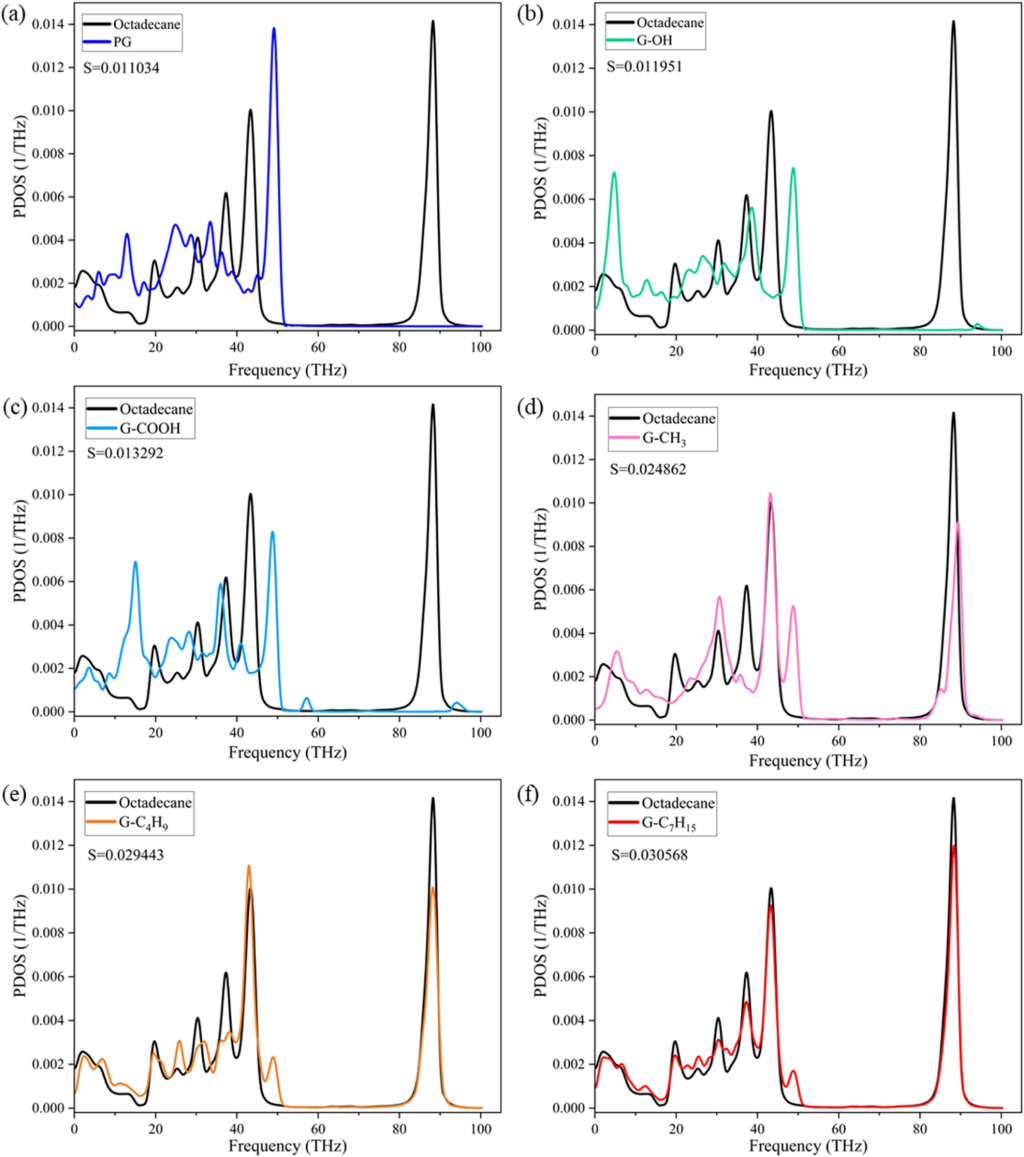
Comparison of PDOS between *n*-octadecane and graphene grafted with various functional groups: (a) PG/OD, (b) G–OH/OD, (c) G-COOH/OD, (d) G-CH_3_/OD, (e) G-C_4_H_9_/OD, and (f) G-C_7_H_15_/OD.

Figure 4 shows the schematic comparison of PDOS and the correlation factor *S* between *n*-octadecane and graphene grafted with various functional groups. As depicted in Figure 4(a), the PDOS peaks of PG at lower frequencies correspond to the out-of-plane phonon modes originating from the flexibility of the graphene basal plane. Additionally, the peak observed at approximately 49 THz represents the in-plane phonon modes, arising from the vibrations of covalent carbon–carbon (C–C) bonds. The PDOS of OD exhibits a peak around 88 THz, attributed to the carbon–hydrogen (C–H) bonds. The high frequency associated with C–H bonds can be attributed to the light hydrogen atoms. It can be seen that both the visually poor PDOS overlap and the small correlation factor *S* of 0.011034 in Figure 4(a) validate the low ITC of PG/OD. Various functional groups improve the overlap of PDOS to varying degrees. However, Figure 4(b,c) shows that the introduction of −OH and −COOH did not significantly improve the overlap of PDOS, which explains the inconspicuous increase in the ITC of G–OH/OD and G-COOH/OD. The comparison results in Figure 4(d–f) reveal that the introduction of alkyl functional groups led to multiple overlapping peaks in both high-frequency and low-frequency regions of PDOS, and the degree of overlap increases with the increase of chain length. The correlation coefficients *S* of G-CH_3_/OD, G-C_4_H_9_/OD, and G-C_7_H_15_/OD are 0.024862, 0.029443, and 0.030568, which are 2.25 times, 2.67 times, and 2.77 times that of PG/OD, respectively. These high degrees of overlap and high *S* values imply a high phonon matching rate, enhancing the interfacial thermal coupling, which explains the prominent improvement in the ITC of G/OD. Both the visually high PDOS overlap and the large correlation factor *S* explain that –C_7_H_15_ significantly improves the ITC of G/OD.

### 3.3 Effect of Covalent Functionalization and Molecular Distribution on the OTC of G/OD

When investigating the effects of covalent functionalization on the interfacial heat transfer of G/OD nanocomposites, the OTC of a selected calculation volume should be taken into consideration, which can be split into interfacial thermal conductance *G*_i_ and matrix thermal conductance *G*_m_. The OTC (G_O_) is calculated as
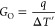
5where Δ*T*′ is the overall temperature difference in the calculation volume. With the graphene sheet as the center, the calculation area containing graphene and a certain volume of *n*-octadecane matrix is selected, and the dimensions of the selected calculation volume are 34.9 Å × 34.5 Å × 30.0 Å.

**Figure 5:**
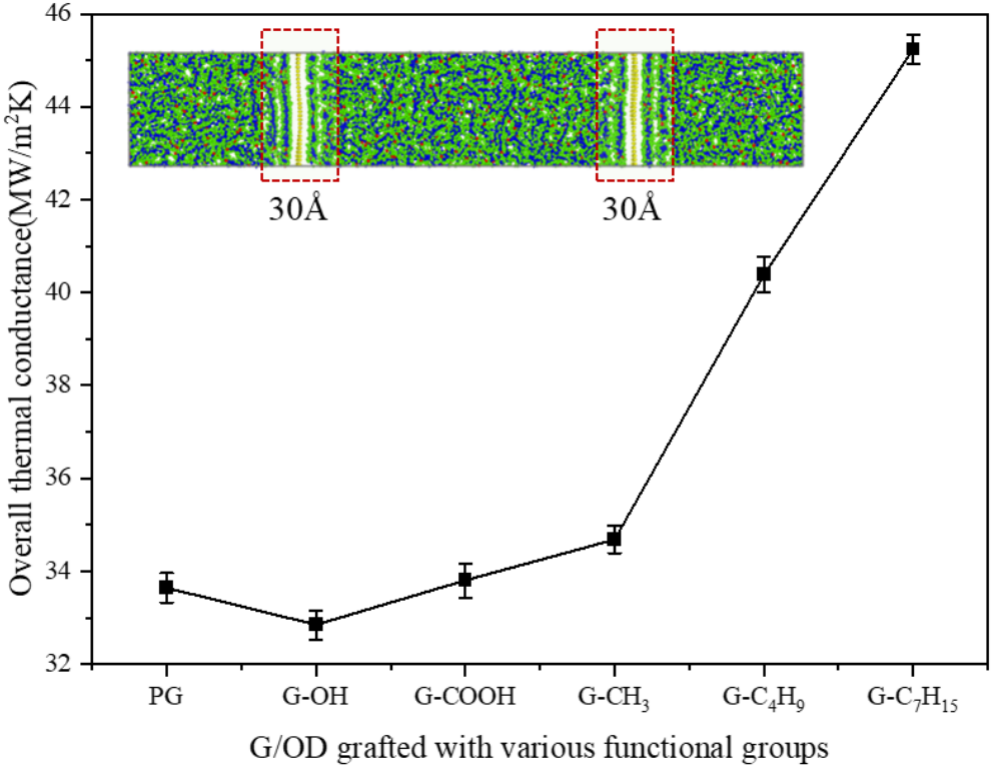
OTC of G/OD grafted with various functional groups.

Figure 5 shows the OTC of G/OD grafted with various functional groups at a graft density of 0.01497 Å^–2^. The calculated OTC of PG/OD is 33.64 MW/m^2^K, which is close to the reported value with a similar selected volume.^51,52^ It is obvious that the effect of the functional group type on the OTC is similar to that on the ITC. The improvement of the ITC contributes to the OTC of the selected volume to a large extent. The most obvious improvement for OTC of G/OD is –C_7_H_15_, which increases from 33.64 to 45.24 MW/m^2^K, followed by –C_4_H_9_, −CH_3_, and −COOH. Unfortunately, the introduction of the −OH functional group instead slightly decreases the OTC.

**Figure 6:**
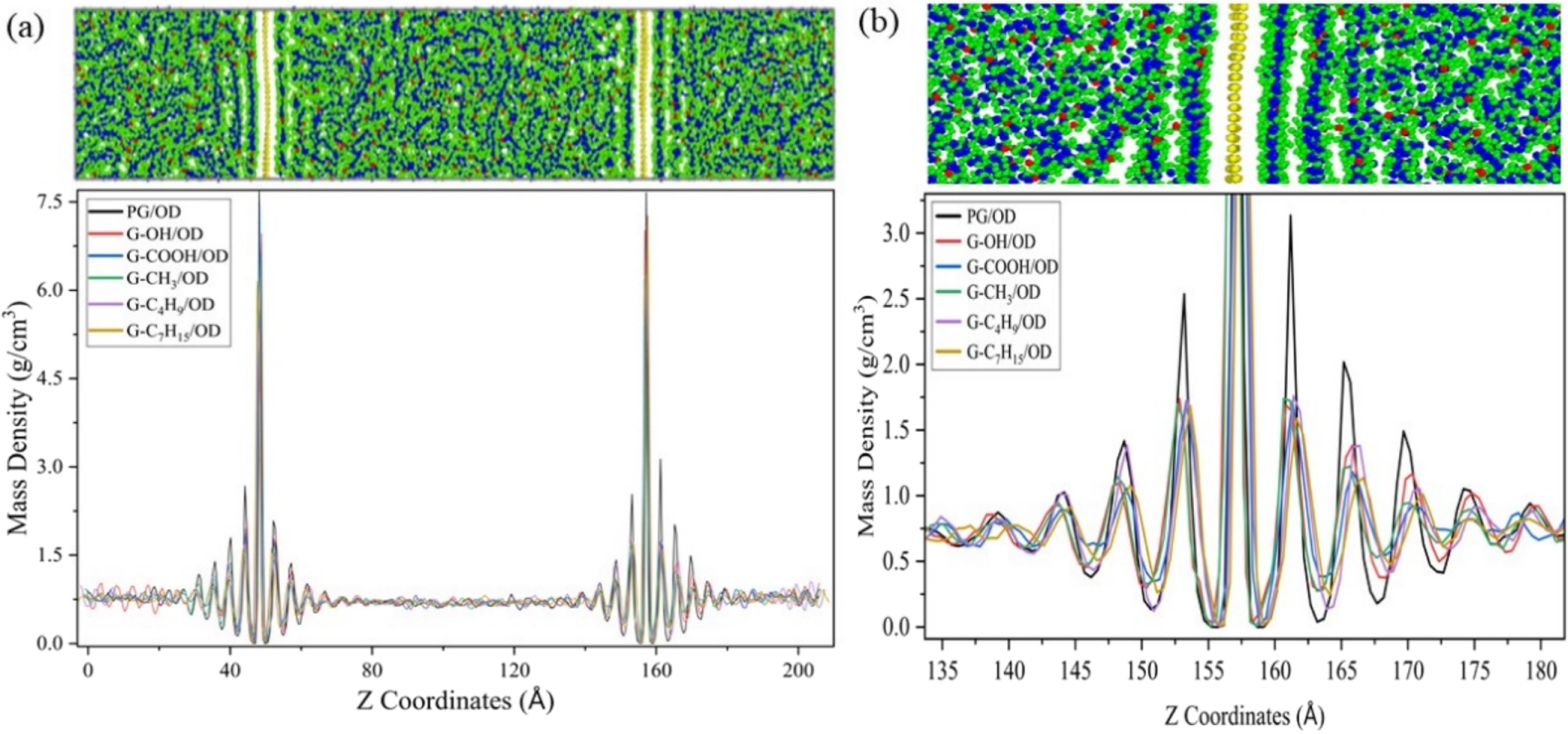
(a) Distribution of the mass density of the G/OD nanocomposite system along the heat flux direction (*z*-axis). (b) Local magnification of (a).

The arrangement of the *n*-octadecane molecular chains can affect the OTC of the G/OD nanocomposites. Therefore, the distribution of the mass density of the G/OD nanocomposite system along the heat flux direction (*z*-axis) is calculated. The mass density ρ is obtained by ρ = *m*/*v*, where *m* represents mass and *v* represents volume. The calculated results are listed in Figure 6(a). The existence of peaks can represent the areas of concentration of atoms, and the two highest peaks represent the two graphene sheets, respectively. Two or three peaks appear on each side of the graphene, and from the graphene interface to both sides, the peaks continuously decay and eventually return to baseline values. According to the density distribution, it can be easily analyzed that the *n*-octadecane molecular chains are uniformly distributed away from the surface of graphene, while under the interaction of graphene and *n*-octadecane molecules, obvious aggregation and stratification occur near the graphene surface. A local magnification of Figure 6(a) is shown in Figure 6(b). As the distance increases, the interaction weakens and therefore the degree of aggregation and stratification decreases. The two layers of n-octadecane molecules near the graphene interface are also known as the near-wall layer or contact layer, which tends to be parallel to graphene.^53^ The distance between the center of the first layer and graphene layer is about 4.0 Å, and the distance between the centers of adjacent *n*-octadecane molecular layers is about 4.2–4.4 Å, which is consistent with a previous study.^53^

Enhancing ITC can be achieved by controlling external conditions, such as applying pressure, controlling temperature, and applying physical fields. These methods aim to increase materials density, achieve close adhesion between materials at the interface, and enhance the van der Waals forces between interfacial materials.^24^ The OTC can be divided into interfacial thermal conductance *G*_i_ and matrix thermal conductance *G*_m_. The interaction between graphene and paraffin molecules results in a local high density and stratification of paraffin molecules near the graphene interface, which subsequently decreases the matrix thermal conductance G_m_ but increases the interfacial thermal conductance *G*_i_.

**Figure 7:**
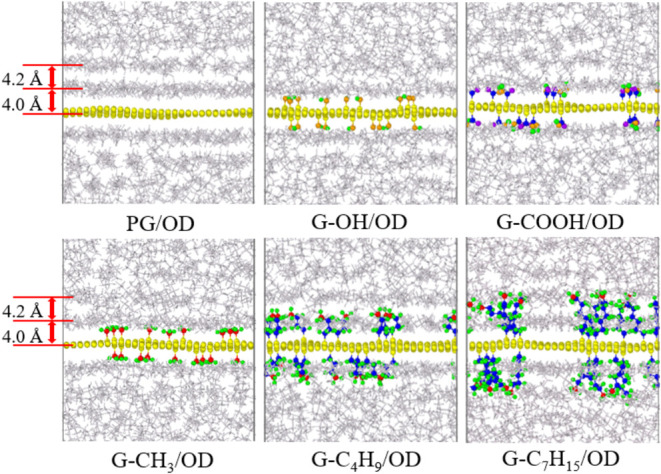
Microstructure near graphene in G/OD systems.

From one perspective, efficient propagation of thermal energy along alkane chains and the stratification phenomenon, where the n-octadecane molecules in the near-wall layer tend to be parallel to the graphene, lead to a decrease in the matrix thermal conductance G_m_ along the direction perpendicular to graphene and thus to a decrease in the OTC. The peaks in Figure 6(b) indicate that the highest degree of stratification is the first layer, followed by the second layer. The introduction of covalent functional groups decreases the degree of stratification. The microscopic structure near the graphene interface is shown in Figure 7. The distance from the center of the first layer to the graphene layer is about 4.0 Å, and the distance between the centers of the first layer and the second layer is about 4.2 Å. The distances from the end atoms in the functional groups to the center of graphene layer are calculated. Due to the natural extension of the functional group chains, the average distances from the end atoms of −OH, −COOH, −CH_3_, –C_4_H_9_, and –C_7_H_15_ to the center of the graphene layer are about 3.4 4.4, 3.6, 7.2, and 10.2 Å, respectively. Combined with the microscopic structure in Figure 7, −OH and −CH_3_ groups approach but do not reach the first layer; −COOH groups reach the first layer; –C_4_H_9_ is close to the second layer; and –C_7_H_15_ reaches the second layer. This implies that the presence of –C_4_H_9_ and –C_7_H_15_ enables heat to propagate more efficiently along the functional group chains from the paraffin matrix, traversing the near-wall layers to reach the graphene. This enhanced heat transfer results in improved interlayer thermal conductance within the near-wall layers, ultimately leading to an elevated OTC.

From another perspective, increasing the density of the paraffin matrix proximal to the graphene interface can intensify the van der Waals forces between paraffin and graphene, subsequently enhancing the heat transfer efficiency at the interface of the graphene/paraffin nanocomposite. Luo and Lloyd^32^ found that when the density of the paraffin matrix increases, the distance between atoms separated by nonbonded or three or more covalent bonds decreases, leading to effectively stronger van der Waals forces. This results in greater spectral overlap and stronger interfacial thermal coupling, which, in turn, improves the ITC. Additionally, the higher local density of the paraffin matrix near the interface leads to more paraffin atoms interacting with graphene, which directly improves the ITC. Therefore, the high local density near the graphene interface improves the ITC, thereby improving the OTC. The introduction of covalent functional groups slightly reduces the local density near the graphene interface, thereby weakening the aforementioned enhancing mechanism. Interestingly, the covalent bond interaction is significantly stronger than the van der Waals interaction. Consequently, heat transfer from covalent functional group chains to graphene is generally much more efficient than through van der Waals interactions, rendering the aforementioned weakening effect less pronounced. However, the visually observed low overlap of PDOS and the small correlation factor *S* indicate that the −OH functional group has a poor enhancement effect on thermal coupling at the G/OD interface. Therefore, the aforementioned weakening effect may be significant in the case of G/OD–OH, potentially explaining why the introduction of the −OH functional group might actually result in a decrease in the level of OTC.

### 3.4 Prediction of TC of G/OD Nanocomposites

It is crucial to associate the ITC with the TC of G/OD nanocomposites that incorporate randomly distributed graphene. To study the effect of covalent functionalization on the TC of G/OD nanocomposites, the EMT by Nan et al.^54^ is adopted to predict the TC of G/PCM. When graphene sheets are randomly dispersed within the n-octadecane matrix, the TC of the G/OD nanocomposites can be expressed as
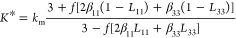
6with
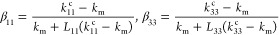
7
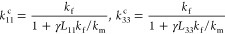
8
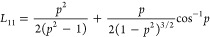
9
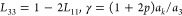
10
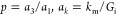
11where K*, *k*_f_, and *k*_m_ are the TC of G/OD, graphene fillers, and *n*-octadecane matrix respectively; *f* denotes the filler volume fraction; *a*_1_ and *a*_3_ denote the width and thickness of graphene, respectively; and *G*_i_ refers to the ITC of G/OD. The value of *a*_*k*_ = *k*_m_/*G*_i_ refers to the Kapitza radius, *p* = *a*_3_/*a*_1_ refers to the aspect ratio of filler. *L_ii_* (*i* = 1, 3) denotes a geometrical factor that depends on the shape of the filler, and *k*_*ii*_^c^ (*i* = 1, 3) denotes the equivalent TC of a unit cell of the nanocomposite along the *i*-axis.

When calculating the TC of G/PCM, the necessary parameters for the EMT are determined based on the aforementioned simulation results. The TC of the OD is 0.169 W/(m·K). The volume fraction of graphene fillers varies from 1 to 9%. The thickness *a*_1_ and width *a*_3_ of the graphene fillers are determined to be 3.35 Å and 5 μm, respectively. The ITC values of G/OD obtained from the simulation results are adopted. The freely suspended graphene exhibits remarkable TC values ranging from 1000 to 5800 W/(m·K).^12^ However, in this study, graphene sheets are embedded in an OD matrix, as the OD matrix acted as substrates supporting both sides of the graphene sheets. The interaction between graphene and the surrounding polymer significantly suppresses phonon transport within graphene. It is found that the TC of graphene supported by the substrate is insensitive to length due to the fact that the ZA phonons are greatly suppressed by the graphene-substrate interaction, and the TC of graphene supported by the substrate at room temperature is approximately 600 W/(m·K).^55−57^ Compared to the TC of graphene itself, the impact of covalent functional groups on the TC of graphene is minimal. It is difficult to accurately determine the TC of graphene grafted with different functional groups that are randomly dispersed in paraffin. Therefore, the value of 600 W/(m·K) is adopted as the TC value of graphene, which is reasonable.^30^

**Figure 8:**
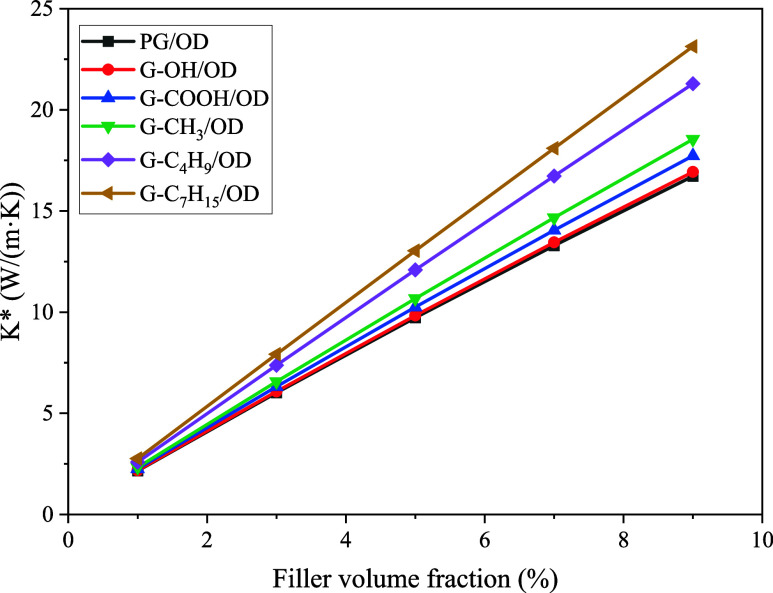
TC of G/OD nanocomposites with respect to the filler volume fraction.

According to the assigned parameters and eqs 6–11, the TC of G/OD nanocomposites concerning the filler volume fraction can be calculated, and the calculated results are shown in Figure 8. It is apparent that the TC of G/OD increases as the filler volume fraction increases. Due to the increase in ITC of G/OD caused by the introduction of covalent functional groups, the TC of G/OD also increases. When the volume fraction of PG increases from 1 to 9%, the TC of PG/OD increases from 2.16 to 16.72 W/(m·K). The TC of G-C_7_H_15_/OD is consistently the highest, followed by G-C_4_H_9_/OD. When the filler volume fraction is 9%, the TC of G-C_4_H_9_/OD and G-C_7_H_15_/OD can reach 21.29 and 23.13 W/(m·K), which are 127 and 138% of the TC of PG/OD, respectively.

## 4 Conclusions

In this paper, the effects of five covalent functional groups (−C_7_H_15_, –C_4_H_9_, −CH_3_, −COOH, and −OH) at different grafting densities (0.00499, 0.00998, and 0.014970 Å^–2^) on the thermal transport properties of the paraffin/graphene interface are investigated using NEMD simulation. The main conclusions are as follows. First, the ITC of G/OD increases with the increase in graft density. Among the various functional groups, −COOH and −OH show no obvious enhancement effect on the ITC of G/OD. In contrast, because the alkyl functional groups can significantly enhance the interface thermal coupling of G/OD, the enhancement effect of alkyl functional groups on the ITC of G/OD is very significant and gradually increases with the increase of alkyl functional groups chain length. When the graft density reaches 0.01497 Å^–2^, the ITC of G-C_7_H_15_/OD and G-C_4_H_9_/OD can reach 2.28 and 1.75 times the ITC of PG/OD, respectively. Second, the effect of –C_7_H_15_, –C_4_H_9_, −CH_3_, and −COOH on the OTC is similar in trend to that on ITC, while the introduction of −OH decreases the OTC. The introduction of covalent functional groups decreases the degree of stratification and the local high density, which may improve the interlayer thermal conductance of the near-wall layer. In addition, based on the calculated ITC results and the EMT, the TC values of G/OD nanocomposites that incorporate randomly distributed graphene are calculated. For the same filler volume fraction, G-CH_7_H_15_/OD consistently has the highest TC, followed by G-C_4_H_9_/OD. When the volume fraction of graphene filler is 9%, the TC of G-C_4_H_9_/OD and G-C_7_H_15_/OD can reach 21.29 and 23.13 W/(m·K), respectively. The above results provide valuable guidance for experimental research on exploiting the potential of TC of paraffin/graphene.
